# A Federated Machine Learning Approach for the Detection and Visualisation of Eye Diseases Using Activation Maps

**DOI:** 10.3390/s26165288

**Published:** 2026-08-20

**Authors:** Filomena Niro, Miriam Di Renzo, Patrizia Agnello, Marta Petyx, Fabio Martinelli, Maurizio Maddalena, Mario Cesarelli, Antonella Santone, Francesco Mercaldo

**Affiliations:** 1 Azienda Sanitaria Regionale del Molise—ASReM, 86100 Campobasso, Italy; 2 Department of Medicine and Health Sciences “Vincenzo Tiberio”, University of Molise, 86100 Campobasso, Italy; antonella.santone@unimol.it; 3 Istituto Nazionale per l’Assicurazione Contro gli Infortuni sul Lavoro, 00144 Roma, Italy; p.agnello@inail.it (P.A.); m.petyx@inail.it (M.P.); 4 Institute for High Performance Computing and Networking, National Research Council of Italy (CNR), 87036 Rende, Italy; fabio.martinelli@icar.cnr.it; 5 IRCCS Istituto Nazionale Tumori, Fondazione Pascale, 80131 Napoli, Italy; m.maddalena@istitutotumori.na.it; 6 Department of Engineering, University of Sannio, 82100 Benevento, Italy

**Keywords:** eye diseases, glaucoma, cataracts, artificial intelligence, federated learning, privacy, expainability, Grad-CAM

## Abstract

Eye diseases, particularly glaucoma and cataracts, are the leading causes of visual impairment, compromising quality of life. Automatic classification of these diseases can lead to more accurate and timely diagnosis, thereby limiting their progression and complications. In recent years, advances in Deep Learning (DL) have shown promising results in the study of images in ophthalmology. However, traditional DL models are based on a centralised approach to data, sharing sensitive patient information and compromising privacy; moreover, the models are often difficult to interpret. In this paper we propose a method aimed to solve the issues of privacy and transparency in decision-making related to eye diseases detection and localisation. As a matter of fact, we consider Federated Learning (FL), an approach based on data decentralisation that enables collaborative learning between different clients and sends only the model weights to the central server. In this way, sensitive patient data are not shared, ensuring security and privacy. With regard to eye disease classification we exploit a Vision Transformer, which allows global relationships within retinal images to be highlighted, improving representation capabilities compared to traditional convolutional architectures. Furthermore, the proposed method also aims to make the model explainable using explainability techniques, in this way we make diagnostic decisions transparent. The experimental analysis shows an accuracy of 0.8480, a precision of 0.8645, a recall of 0.8477, showing the effectiveness of the proposed method on eye disease detection.

## 1. Introduction

The eye is the organ of the human body specialised in perceiving light and transforming light signals into nerve impulses. In order to ensure clear and functional vision, the eye is made up of several important anatomical structures. In particular, the outermost part includes the cornea, a transparent membrane responsible for the passage of light and the focusing of images. The iris is the coloured part of the eye and it regulates the amount of light entering through the pupil. In the innermost part of the eye is the lens, whose elastic structure allows humans to see both near and far, and finally the retina, a nerve tissue rich in photoreceptors responsible for converting light signals into electrical impulses. Alterations in each of the structures described above cause complex eye diseases that can lead to vision loss. The most common diseases are glaucoma and cataracts. According to numerous studies, glaucoma is an eye disease characterised by increased intraocular pressure that can progressively damage the optic nerve. This damage is often silent in the early stages and manifests itself as a gradual reduction in the field of vision, eventually leading to blindness in more advanced cases. Cataracts, on the other hand, manifest themselves in the form of clouding of the lens, which gradually loses its transparency. The main consequences are: blurred vision, increased sensitivity to light and difficulty in perceiving colours. Unlike glaucoma, cataracts can be treated through surgery with the replacement of an artificial lens. Early and timely diagnosis can certainly reduce the risk of the disease progressing. In recent years, biomedical images have been analysed using Artificial Intelligence (AI) techniques; in this context, Deep Learning (DL) shows interesting potential in predicting the disease. This is the context for the study conducted by [1], which involved the use of seven architectural configurations comprising Vision Transformers (ViTs), consolidated Convolutional Neural Networks (CNNs) and hybrid models in three different classification scenarios. The dataset used, consisting of 456 OCTA images, was tested on different networks, which allowed DenseNet121 to be identified as the optimal architecture, achieving an accuracy of 0.79 and ROC-AUC of 0.89. Another important contribution was made by [2], who conducted a study on the severity of Diabetic Retinopathy (DR) using Federated Learning (FL). In order to identify the signs of Diabetic Retinopathy, the authors combined the aggregation algorithm known as Federated Averaging (FedAvg) with the median of the categorical cross-entropy with loss function and achieved an accuracy of 0.98. The detection of glaucoma using FL techniques was also addressed by [3], who conducted a study with seven clients located in hospitals in Hong Kong, Singapore and the USA on a set of local optic disc volumetric data. The weights relative to the results obtained were then sent to the central server and aggregated using the Federated Proximal (FedProx) algorithm. The analysis conducted by [4] involved the creation of a binary classification system for glaucoma detection. In this context, federated strategies included the adoption of FedAvg, FedProx, Federated Learning on Non-IID Features via Local Batch Normalization. (FedBN), and Federated Yogi (FedYOGI) algorithms. In particular, the experiment conducted using FedProx achieved an accuracy of 0.76 and a precision of 0.69. Although FL mitigates the risks associated with data centralisation, recent literature has shown that simply sharing model parameters does not guarantee absolute privacy. In this regard, ref. [5] highlighted the risk of information leakage; in such cases, sensitive data can be reconstructed through gradient analysis via model inversion or membership inference attacks. To counter these vulnerabilities, advanced defence mechanisms can be adopted, such as differential privacy and data encryption techniques, aimed at preserving the integrity of patient information without unduly compromising model performance. Starting from these considerations, in this paper we propose the adoption of FL using ViT with explainability techniques for the detection of cataracts and glaucoma within a series of fundus images. The use of FL enables data sharing while maintaining strict privacy and security standards. Classification is performed by a ViT, chosen for its ability to model global relationships within the image. Unlike traditional CNNs, ViT divides the image into several patches of fixed size, which are then transformed into vector embeddings. The explainability techniques adopted, such as Gradient-weighted Class Activation Mapping (Grad-CAM), allow the regions of the image that are most influential for classification to be identified. Grad-CAM allows us to assess whether the model correctly focuses on clinically interesting areas such as optic nerve damage in glaucoma and lens opacification in cataracts. In the next section, we present a description of the methodology adopted for the classification of eye diseases. Section 3 defines the experimental setup and discusses the results obtained. The state of the art is discussed in Section 4 and, in the last section, conclusion and future research directions are drawn.

## 2. The Method

**Figure 1:**
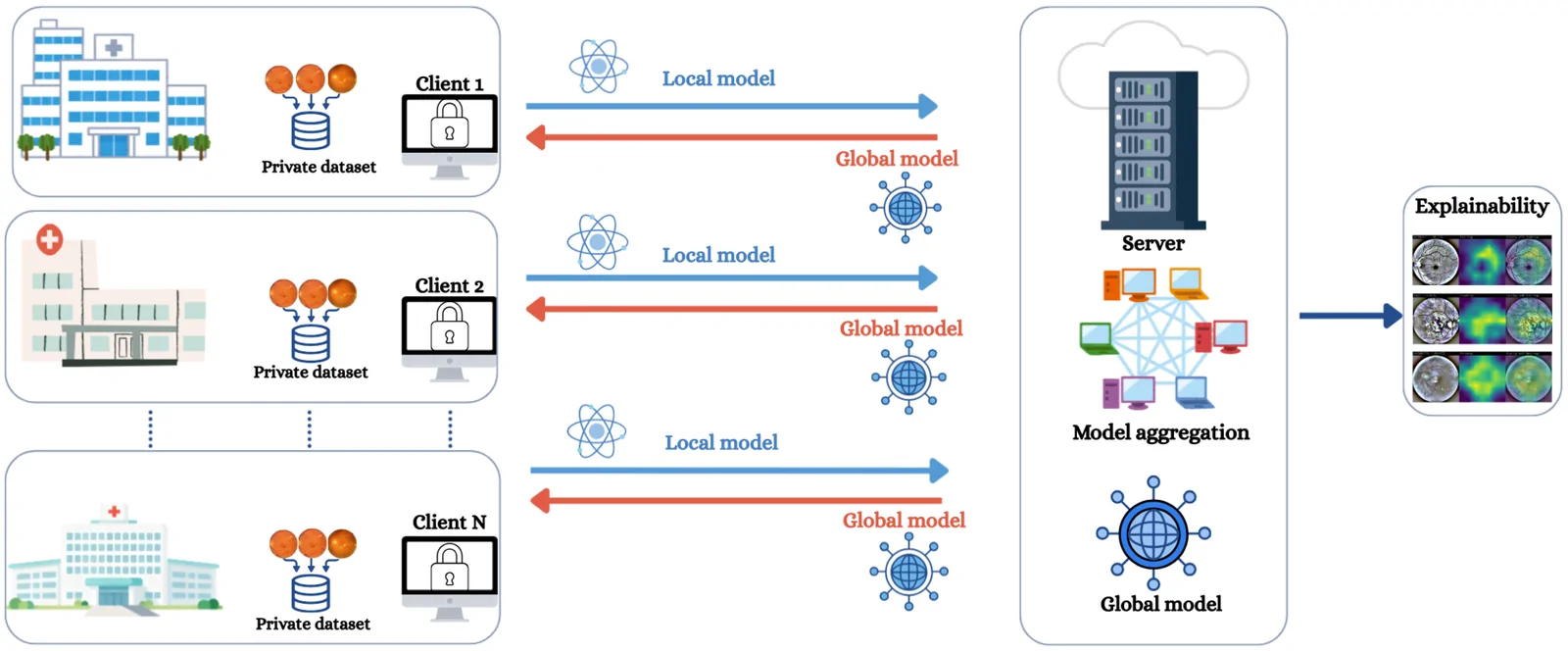
Federated Learning process adopted by the proposed method.

In this section, we illustrate the proposed method for the automatic classification of ophthalmic images for eye disease detection, distinguishing between three clinical conditions: normal eye, cataract, and glaucoma. The proposed method considers the integration of FL, ViT, and explainability techniques. The method consists of several steps, as illustrated in Figure 1.

**Figure 2:**
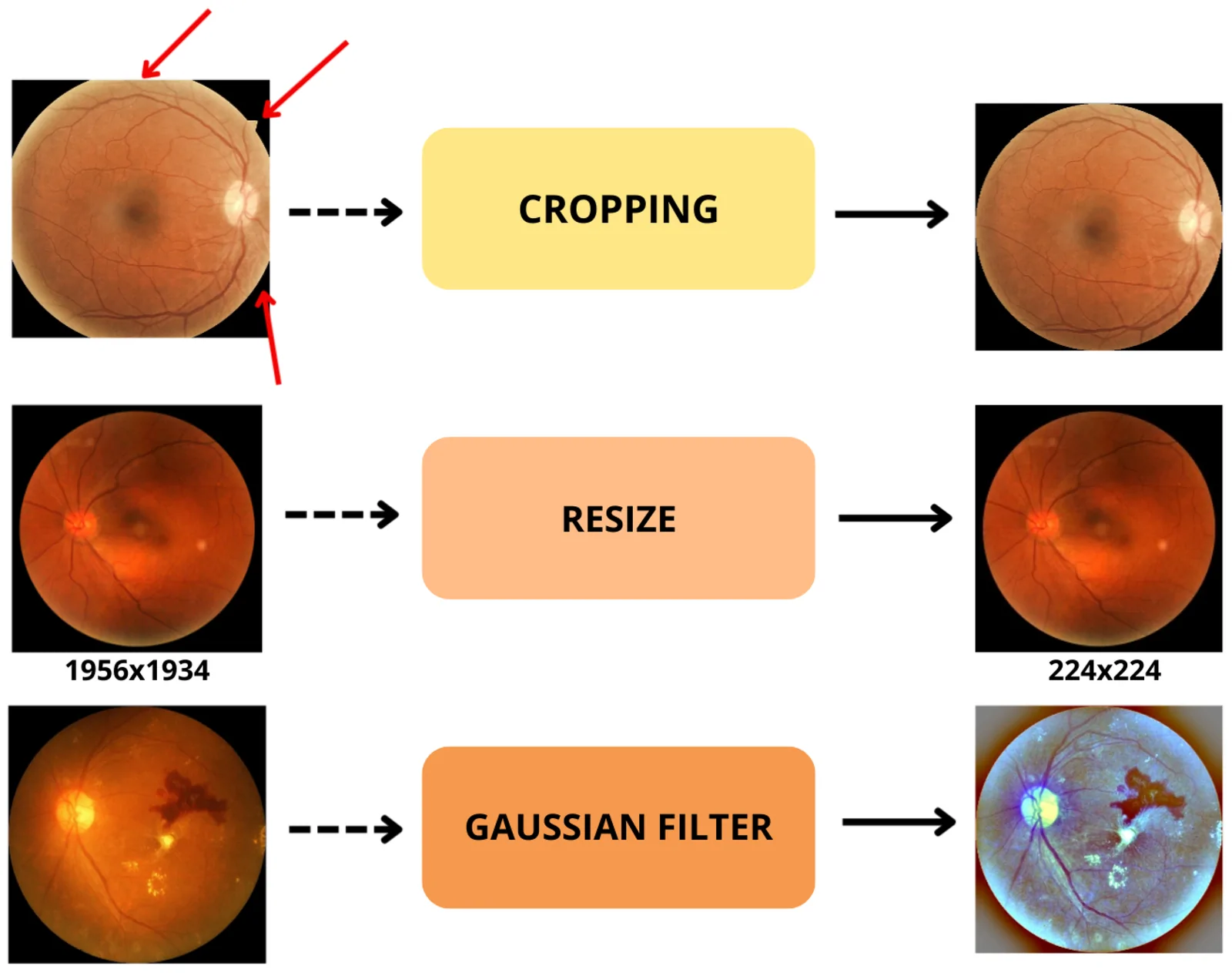
Preprocessing stages. The pre-processed images are shown on the left of the figure; the post-processed images are shown on the right.

The first step involved the acquisition and preprocessing of images. The data were obtained from a public dataset, and the preprocessing phase illustrated in Figure 2 was necessary to make the images homogeneous and suitable for model input. As illustrated in Figure 2, cropping was performed: this operation reduced possible biases in the images due to the presence of dark edges and areas outside the retinal disc. Subsequently, all images underwent a resizing process set at 224 × 224 pixels to ensure compatibility with the input resolution of the pretrained ViT backbone. Finally, to reduce lighting variables, a Gaussian filter was applied, making the images more homogeneous.

Once the real-world dataset was collected, the acquired images had to be preprocessed in order to standardize them, regardless of the imaging device or acquisition settings used. The preprocessing pipeline consisted of two main steps: the application of a Gaussian filter and a subsequent cropping operation designed to centre the eye images.

The Gaussian filter was implemented by convolving each image with an impulse response defined as a centred, normalized Gaussian function with unit sum. Since the Gaussian kernel is separable, the convolution can be efficiently performed by applying the filter along rows and then along columns (or vice versa). It is worth noting that the Fourier transformation of a Gaussian is still a Gaussian; therefore, the Gaussian filter behaves as an approximation of a low-pass filter. For this reason, its application effectively suppresses high-frequency noise while preserving the low-frequency content.

Listing 1 reports the Python code developed to preprocess the eye images through Gaussian filtering and cropping operations.

**Listing 1.** Code snippet related to image preprocessing.

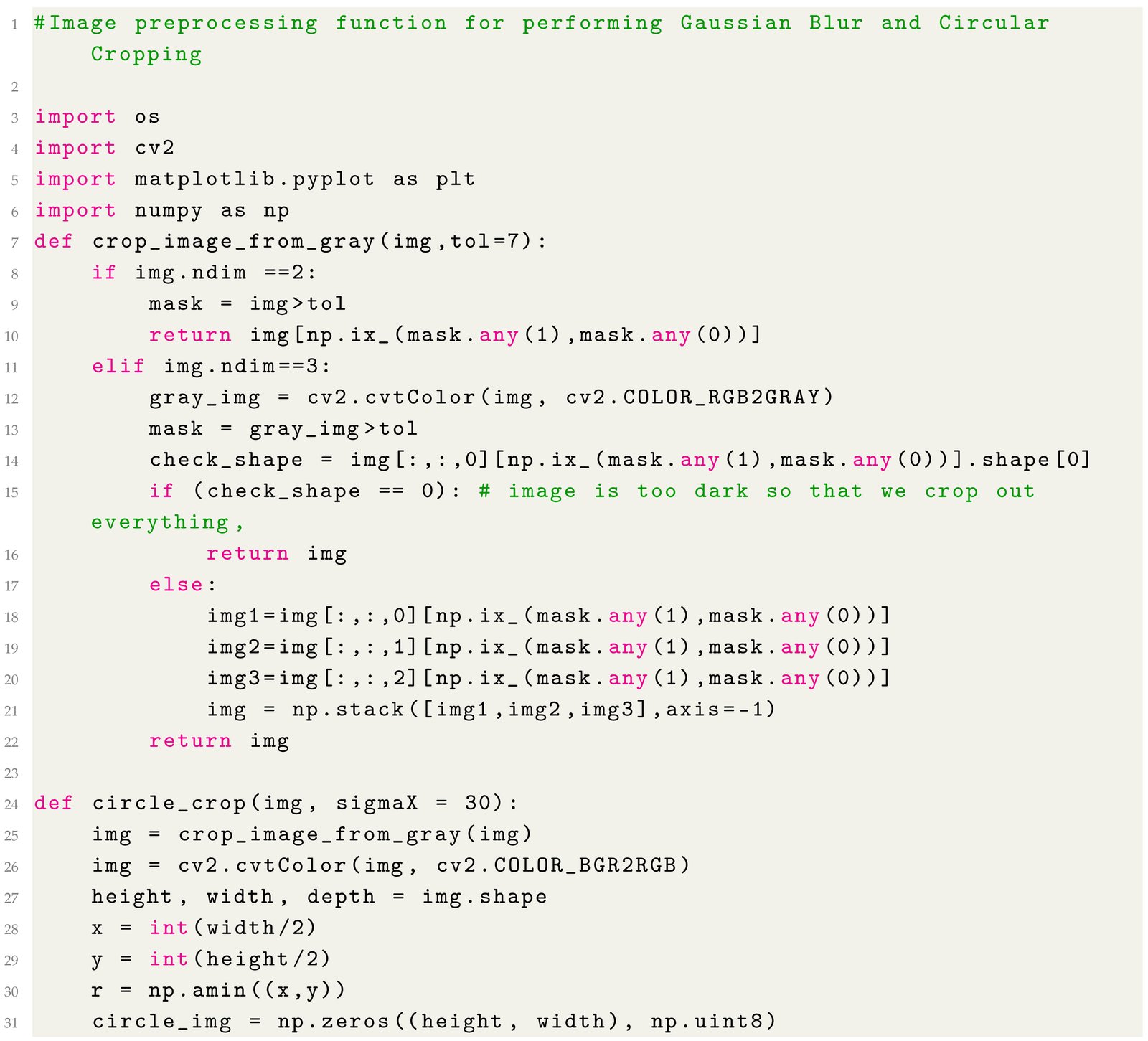



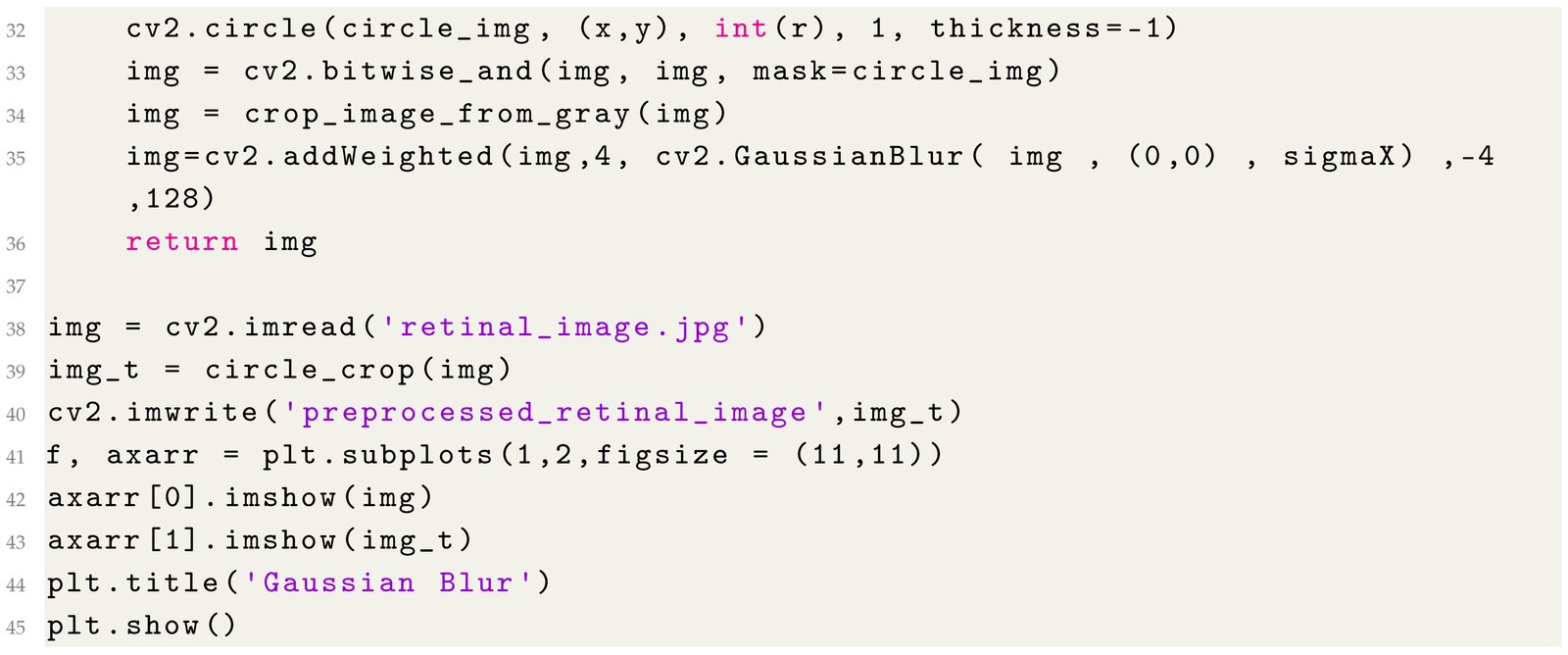



Once the preprocessing phase was complete, the images were divided into three classes, normal, cataract, and glaucoma, which were then divided into training, validation, and test sets. Subsequently, as shown in Figure 1 the model was trained in a federated manner, i.e., in distributed mode without the data leaving the local sites. This process is divided into iterative cycles, or federated rounds. As a first step, the central server initialises a global model that is sent to the clients, then each client trains the model using only its own private dataset. Once local training is complete, only the updated model weights are transmitted to the server. Specifically, in order to optimise the central model, the weights of the updated models are switched for each iteration, including the learning rate, batch size, aggregator and the number of epochs considered. At this point, the server aggregates the parameters using two possible algorithms: FedAvg or FedProx. This results in a new version of the global model, which is redistributed to the clients for the next round. This approach allows us to exploit the heterogeneity of multi-centre data, increasing the robustness of the model and maintaining high standards of security and privacy. Classification is performed by a ViT, chosen for its ability to model global relationships within the image. Unlike traditional CNNs, ViT divides the image into several patches of fixed size, which are then transformed into vector embeddings. These embeddings are integrated with positional encoding information and processed by a series of Transformer encoders, capable of learning long-range dependencies. The Transformer output is finally sent to a classification head that generates the probability of belonging to the three diagnostic classes under consideration. The ViT architecture is particularly useful in medical image analysis, where patterns of clinical interest can be distributed across macro-zones. In a federated context, ViT is trained locally on each client during each FL round. Clients train local models using a DL architecture; in particular, in this work we adopted the ViT model [6], which divides input images into fixed-size patches and processes them as sequential tokens through a Transformer encoder. This model captured both local and global dependencies within OCT images, enabling robust detection of eye conditions. Each client initialises its local training using the current global model received from the central server and updates the ViT classifier using its own local dataset.

Listing 2 presents the Python code snippet corresponding to the ViT model used in our framework.

**Listing 2.** Code snippet related to the ViT model.

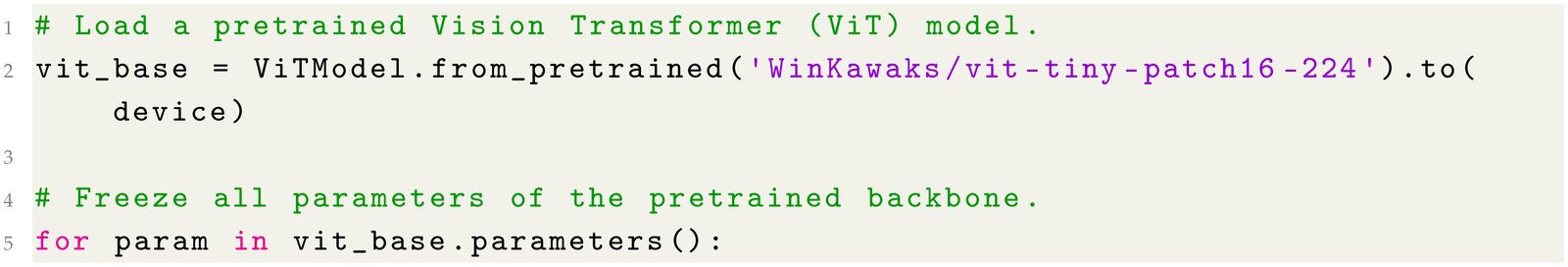



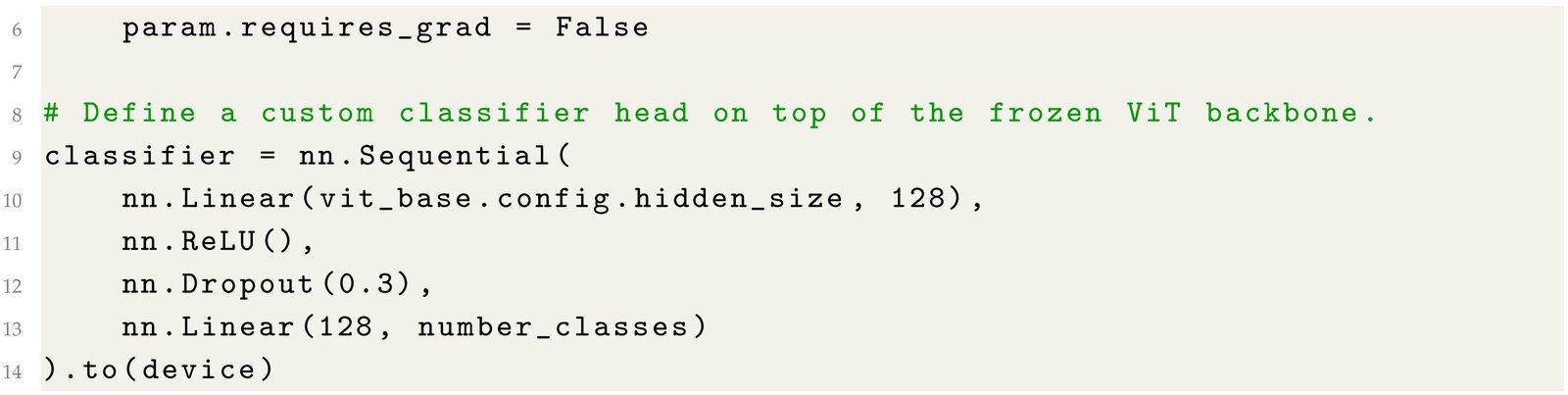



As shown in Listing 2, we rely on the ViT architecture, a Transformer-based model designed for image classification tasks [6]. In detail, in this paper, we resorted to the pretrained WinKawaks/vit-tiny-patch16-224 model obtained from the Hugging Face transformers library (https://huggingface.co/WinKawaks/vit-tiny-patch16-224 accessed on 3 Septemeber 2025), which is freely available for research use. Exploiting a pretrained model i.e., one initialised with weights learned from large-scale image datasets, ensures that the backbone provides rich and generalisable visual representations. The decision to freeze the ImageNet-pretrained Vision Transformer backbone and train only the classification head was primarily motivated by the limited size of the imaging dataset used in this study. Fine-tuning the entire network, or even deeper Transformer blocks, substantially increases the number of trainable parameters, which can easily lead to overfitting when only a relatively small number of labelled medical images is available. In contrast, the frozen backbone acts as a robust generic feature extractor, while the classification head adapts these learned representations to the target task. Furthermore, freezing the backbone significantly reduces computational cost and training time while providing stable convergence.

The code snippet reported in Listing 2 encompasses the following components:

- Feature Extraction with ViT: The pretrained ViT is loaded and all its parameters are frozen. As a consequence, the ViT backbone is used exclusively as a feature extractor, and its weights remain unchanged throughout training. This transfer learning strategy is commonly adopted when the available dataset is limited or when pretrained visual features are sufficiently expressive for the downstream task.
- Custom Classification Head: A fully connected classifier is added on top of the frozen backbone. It consists of:
  1. A Linear layer projecting the ViT hidden representation into a 128-dimensional feature space.
  2. A ReLU activation function for non-linearity.
  3. A Dropout layer with a dropout probability of 30% to reduce overfitting.
  4. A Final Linear layer mapping the 128 features to the number of output classes (three: normal, cataract, glaucoma).

This architecture allows the proposed method to exploit the representational capacity of the ViT structure, for the classification of eye disease detection from fundus images.

After the local ViT training, each client sends its model updates to the central server, which produces a new global model via federated aggregation. The proposed method considers FedAvg and FedProx aggregation strategies.

In particular, FedAvg computes the global model by averaging client parameters proportionally to their dataset sizes:

(1) $$\theta_{t+1}=\sum_{k=1}^{K}\frac{n_{k}}{n}\theta_{t}^{k},$$

where $\theta_{t}^{k}$ denotes the model of client *k*, $n_{k}$ is the number of its local samples, and $n=\sum_{k=1}^{K}n_{k}$ is the total number of training samples.

FedProx extends FedAvg by incorporating a proximal term into the local optimisation objective to address data heterogeneity and partial participation. The client-side objective becomes:

(2) $$\min_{\theta }\;f_{k}\left(\theta \right)+\frac{\mu }{2}{∥\theta -\theta_{t}∥}^{2},$$

where $f_{k}\left(\theta \right)$ is the local loss, $\theta_{t}$ the global model, and μ a regularisation parameter controlling the strength of the proximal term.

As introduced, the proposed method also integrates explainability techniques to ensure that the model’s decisions are interpretable. In particular, explainability techniques such as Grad-CAM were used, which allow the regions of the image that are most influential in the prediction to be visualised. In the following, we describe the various steps required to apply Grad-CAM to ViT, which differs from the classic CNN approach in that it requires the reorganisation of patch sequences (tokens) into a 2D spatial map:

- Forward Pass and Target Layer Selection: The image is passed through the ViT to obtain predictions. In this scenario there are two main layers that are responsible for the final predictions: a Dense Layer, which reduces the size of the data so that it matches the number of desired classes (in this case, three); and a Softmax Layer, which is responsible for the probabilities returned as output.
- Calculation of Gradients (Backward Pass): At this stage, the gradient of the target class score is calculated with respect to the outputs of the tokens in the selected Transformer block.
- Global Average Pooling of Gradients: Once the gradients have been calculated for each patch/token, they are spatially averaged to obtain a weight for each feature channel.
- Token Reshaping: Since the output of the ViT is a sequence, it is necessary to remove the CLS token and reshape the sequence into a 2D grid to reconstruct the image.
- Weighted Combination and ReLU: The remodelled feature maps are multiplied by the weights calculated in step 3 and added together. The ReLU function is applied to consider only those features that have a positive influence on the target class.
- Upsampling and Visualisation: The resulting activation map, which is smaller in size, is enlarged again to the original image resolution and superimposed on it to highlight the “hot” areas.

The advantage of using activation maps is that they allow us to verify whether the model correctly focuses on clinically relevant areas, such as lens opacity in cases of cataracts or disc alteration for glaucoma.

The ColorJet colour map was used for the visual representation of the activation maps. This map is characterised by a transition of colours ranging from deep blue to cyan, green, yellow, and finally red. In terms of explainability, these colours indicate the following:

- Blue: The model pays little or no attention to this area; these regions have minimal contribution to the model’s decision.
- Cyan/green: Regions of moderate importance, representing areas of medium relevance for the model’s reasoning.
- Yellow/red: Highly influential regions that strongly contribute to the decision of the model, with red highlighting the most activated areas.

The use of explainability increases the transparency of the proposed method, thereby increasing the confidence of clinical specialists. Therefore, the proposed method aims to combine FL for the protection of sensitive data, ViT for effective image representation, and explainability to ensure clinical interpretability. This allows for the development of an accurate classification system that respects privacy requirements and the transparency required in the clinical context.

## 3. Experimental Analysis

In this section, we describe the experimental analysis aimed at demonstrating the effectiveness of the proposed method in detecting and localising eye diseases.

We considered a freely available dataset (for research purposes) obtained from a Kaggle web repository (https://www.kaggle.com/datasets/gunavenkatdoddi/eye-diseases-classification accessed on 15 November 2025) composed by real-word retinal images with their related label.

The analysis involved the use of a public dataset containing normal retinal images, cataracts, and glaucoma.

- Normal eye: Characterised by a healthy retina.
- Eye with cataract: Opacification of the lens is observed.
- Eye with glaucoma: Retina where there is a change in the optic disc related to the loss of visual field.

### 3.1. Dataset

The dataset used comprised 1293 images, as shown in Table 1. It was divided into training, which included the images needed to train the model; validation, which included the images needed to validate the models examined; and finally testing, which included the images used to test the robustness of the models in image classification. In the proposed work, each client simulated the behaviour of a separate hospital, with each one actively participating in every local round. It should also be noted that the number of samples per class analysed and assigned to each client was equal to 10 per cent of the total number of images in the dataset.

### 3.2. Experimental Configuration

The experiments were conducted using the Google Colab web platform, with a Python 3 execution environment. The ViT-Winkawaks/vit-tiny-patch16-224 model, belonging to the ViT family, was used for the classification task. The experimental phase was conducted by systematically varying several hyperparameters, as shown in Table 2. In particular, the number of clients was considered, representing the number of institutions involved in the federated training process. In addition, the number of epochs was varied, i.e., the number of complete iterations on the local dataset performed by each client during federated training. Another parameter we considered was the number of federated rounds, which represents the number of global aggregation iterations performed by the central server. Finally, the impact of the Learning Rate (LR) was evaluated, a parameter that controls the step size with which the optimisation algorithm moves in the parameter space with the aim of minimising the loss function. In the various configurations, the batch size, that is the number of data points the model processed in a single iteration before updating its parameters, was set to 32.

### 3.3. Experimental Results

**Figure 3:**
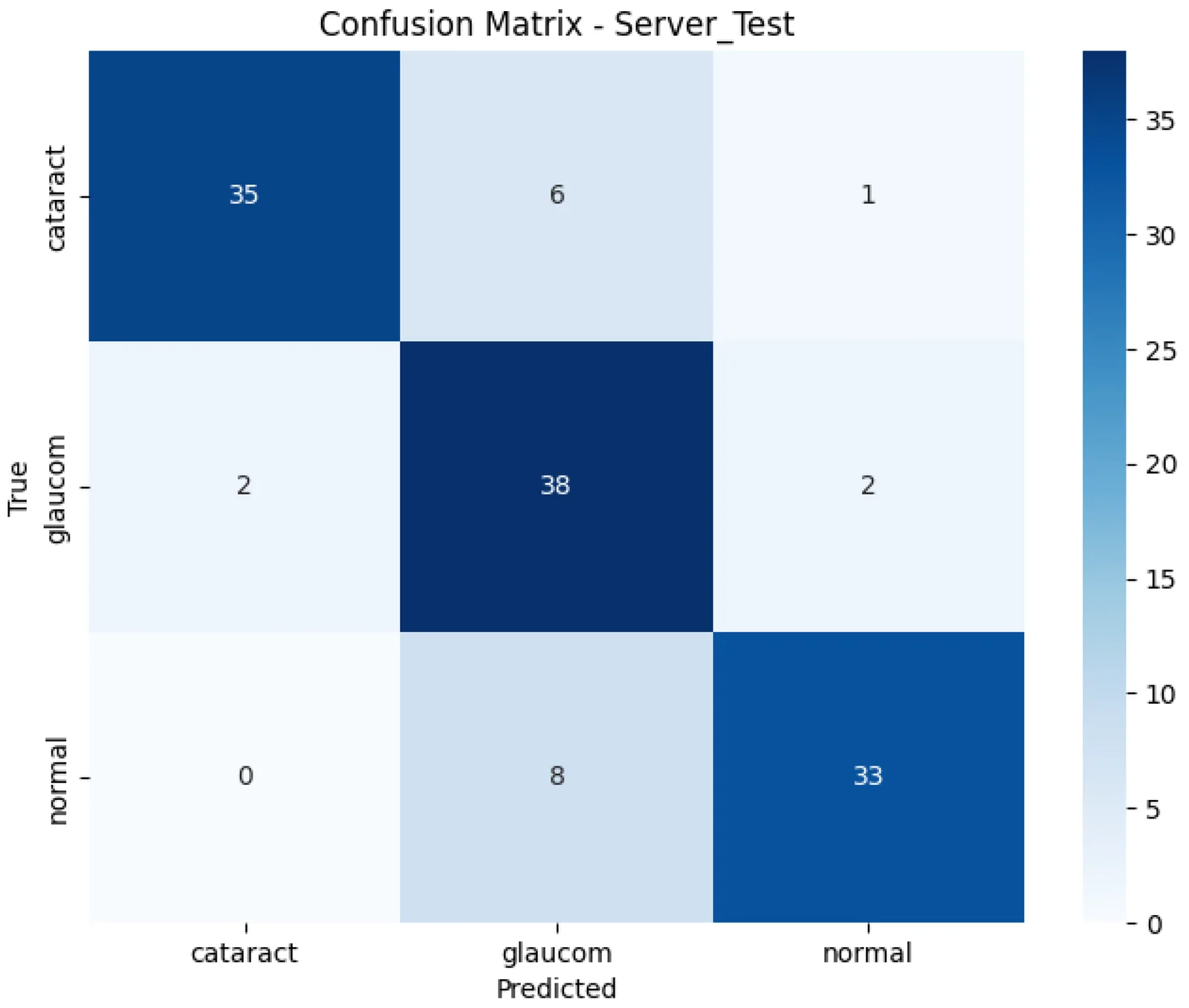
Confusion matrix connected to Experiment 3.

**Figure 4:**
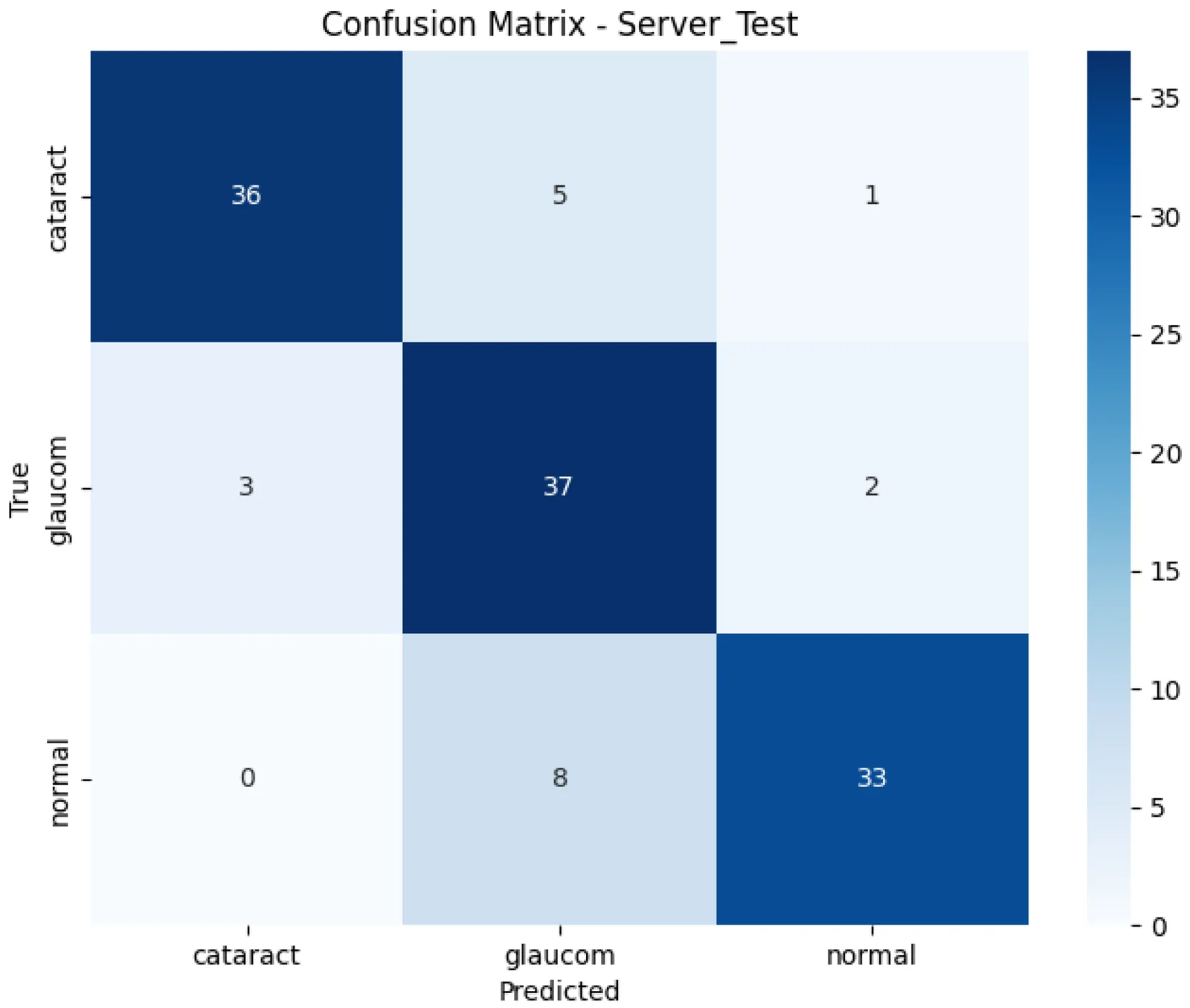
Confusion matrix connected to Experiment 10.

Table 3 shows the performance obtained in the ten experimental configurations evaluated. As shown from Table 3, Experiments 3 and 10 achieved the best results, both reaching an accuracy of 0.8480. By analysing the hyperparameters of the two experiments shown in Table 2, Experiment 3 used FedProx, while Experiment 10 considered FedAvg. Although accuracy and recall were the same in both experiments, Experiment 3 exhibited slightly higher performance in terms of precision and AUC, so the FedProx model achieved a better balance between precision and recall by increasing reliability in discriminating between the different eye diseases. In contrast, Experiment 10 achieved a significantly lower loss. Although a low loss indicates greater proximity between predicted and actual values during training, it is not necessarily indicative of better generalisation ability. In this context, the lower loss obtained in Experiment 10 could indicate an adaptation to the data distribution during training, while the use of the FedProx aggregator in Experiment 3, supported by both the proximal regularisation index and a greater number of aggregation rounds, favoured a more stable and generalisable convergence. Then, experiments were conducted under centralised conditions and with a non-IID data distribution, whilst keeping the hyperparameters from Experiment 3 set under IID conditions fixed. In the proposed study, a Dirichlet distribution with a concentration and diversification factor alpha of 0.3 was adopted in order to generate non-IID data partitions. This choice made it possible to simulate a realistic scenario in which customers represent the majority of the classes comprising the dataset under consideration but with highly asymmetric frequencies. The distribution of the classes under analysis for each client is defined in the Table 4. The comparative analysis shown in the Table 5 reveals crucial insights into the model’s robustness. Introducing a non-IID distribution, which reflects the actual variability in disease prevalence across different hospitals, results in a natural reduction in performance. Indeed, the accuracy of the federated model decreases by 4.8 percentage points when moving from the IID scenario to the non-IID scenario. This result demonstrates that, even though data divergence has a significant impact, the proposed framework manages to partially mitigate the negative effects of heterogeneity. Whilst it does not outperform the centralised baseline model, the federated approach proves to be a valid compromise, offering competitive performance whilst ensuring genuine protection of sensitive data privacy. Subsequently, a comparative analysis of the respective confusion matrices was performed. In particular, the confusion matrix shown in Figure 3 is related to Experiment 3, while the one in Figure 4 corresponds to Experiment 10. Both experiments demonstrate solid classification capabilities. In Experiment 3, there are 36 correct classifications for cataract, 37 for glaucoma, and 33 for normal. Experiment 10 shows comparable results, with 35, 38, and 33 correct predictions. These small differences confirm that both aggregation strategies achieve similar predictive accuracy. A more detailed examination of the error patterns reveals subtle differences; as a matter of fact, cataract cases are better recognised in Experiment 3, while Experiment 10 better classifies glaucoma cases. However, in both experiments, eight samples belonging to the normal class are misclassified as glaucoma: this means that there is a systematic overlap between the representations of the normal and glaucoma classes for both models. Moreover, the confusion matrix of Experiment 3 shows a slightly more balanced distribution of errors, with no obvious bias towards specific classes. This behaviour is consistent with the expected stabilising effect of FedProx in the presence of non-IID data. Conversely, despite the lower loss observed in Experiment 10, no substantial improvements in the error structure are detected. Considering the results as a whole, it emerges that although the two experiments have equivalent accuracy and recall, the use of the FedProx aggregator provides improvements in terms of precision and AUC and a more regular misclassification. This evidence supports the selection of Experiment 3 as the overall most robust configuration, particularly with regard to generalisation and discrimination between classes.

### 3.4. Explainability Analysis

**Figure 5:**
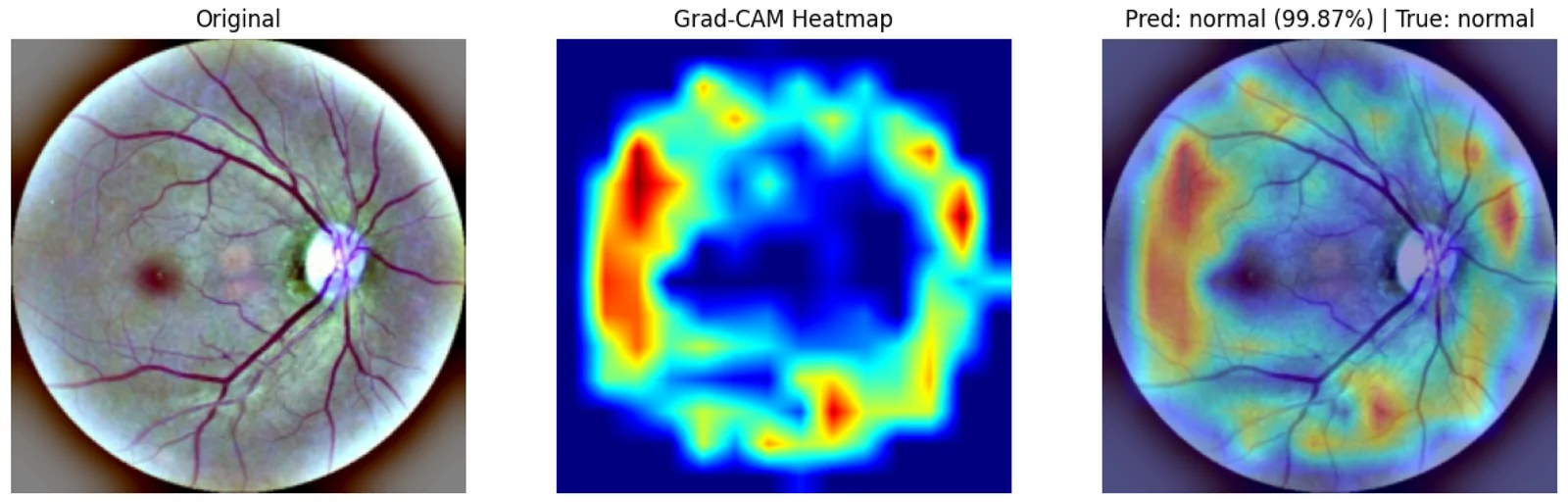
Grad-Cam related to an image belonging to the normal class obtained with the model of Experiment 3.

**Figure 6:**
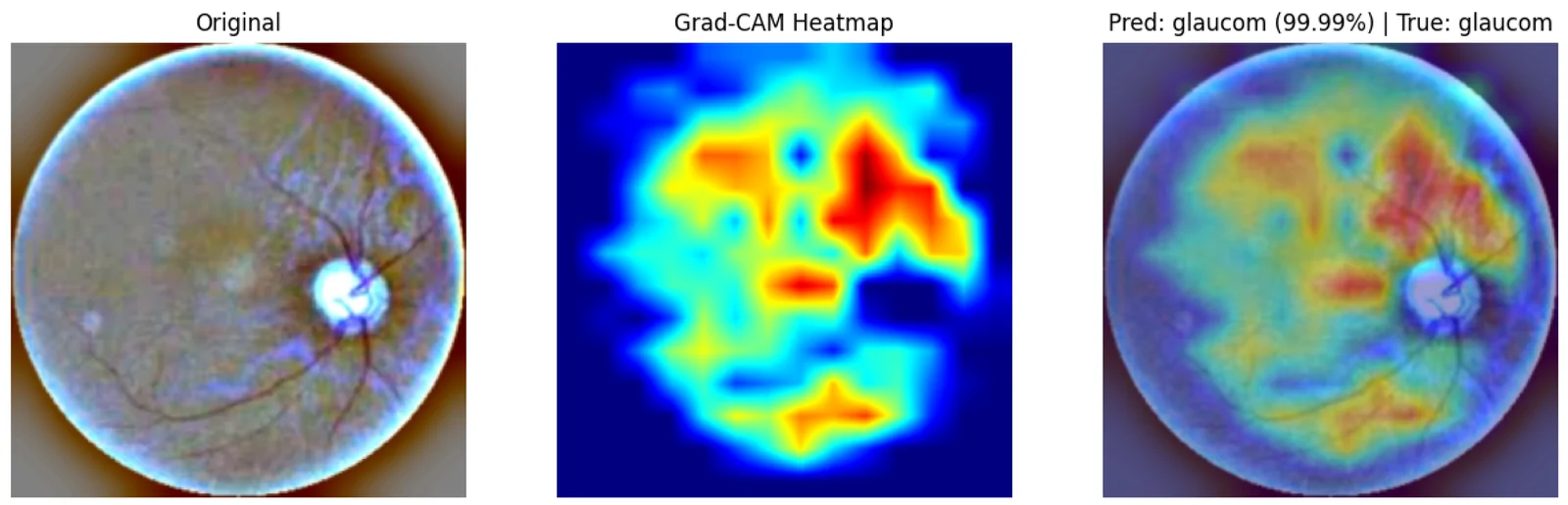
Grad-Cam related to an image belonging to the glaucoma class obtained with the model of Experiment 3.

**Figure 7:**
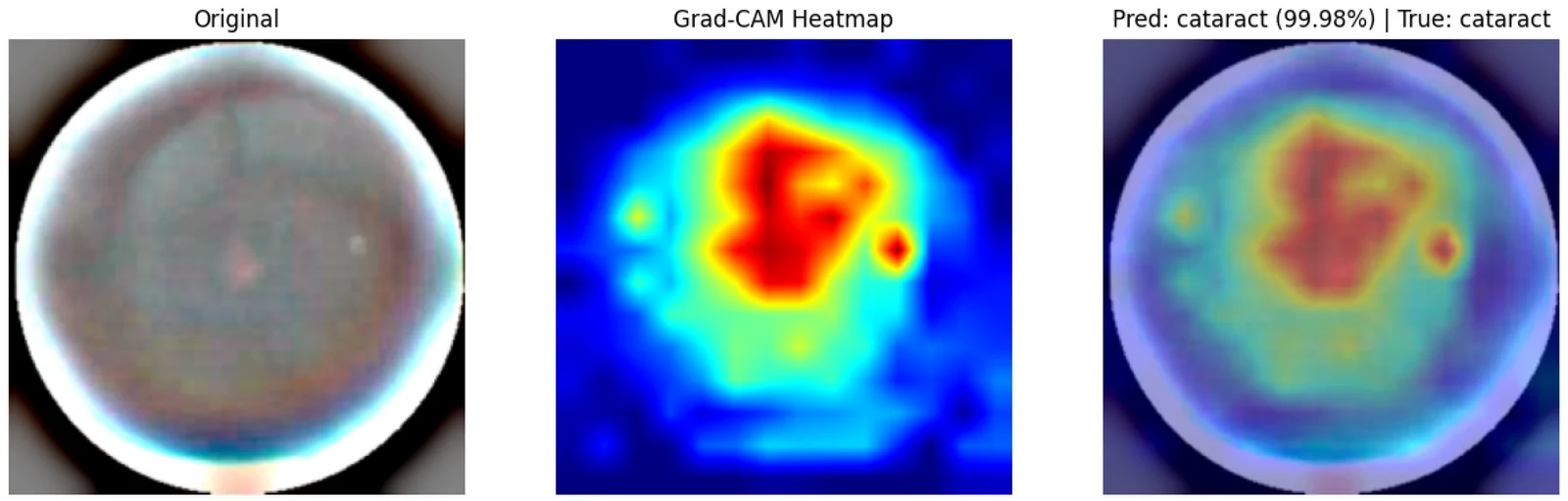
Grad-Cam related to an image belonging to the cataract class obtained with the model of Experiment 3.

**Figure 8:**
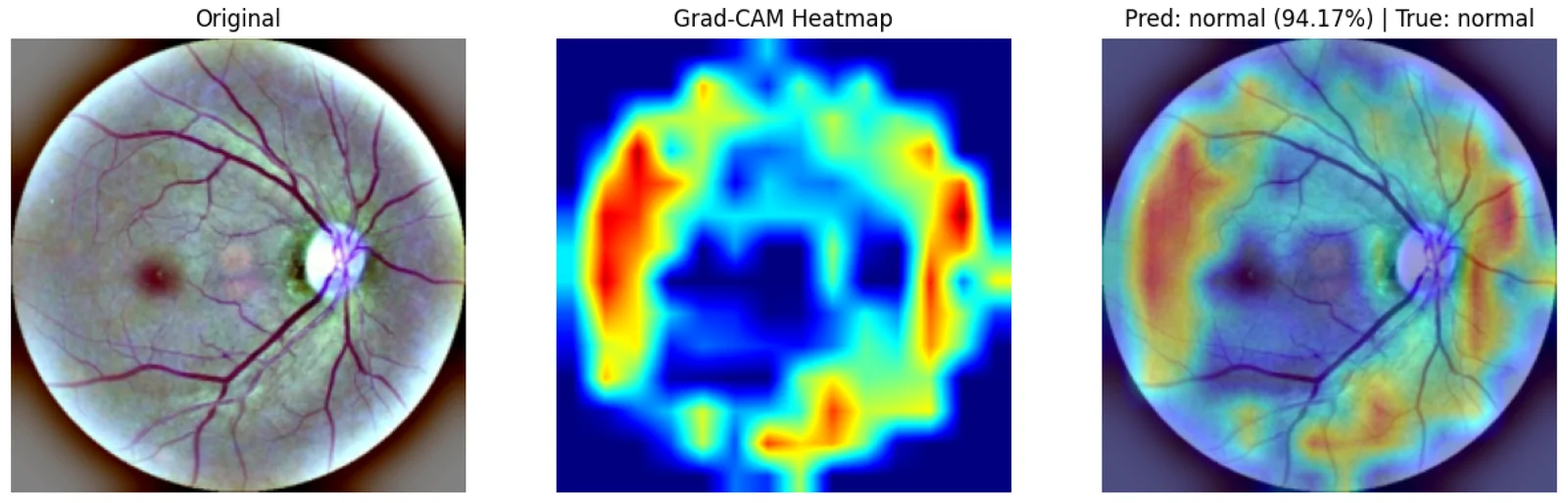
Grad-Cam related to an image belonging to the normal class obtained with the model of Experiment 10.

**Figure 9:**
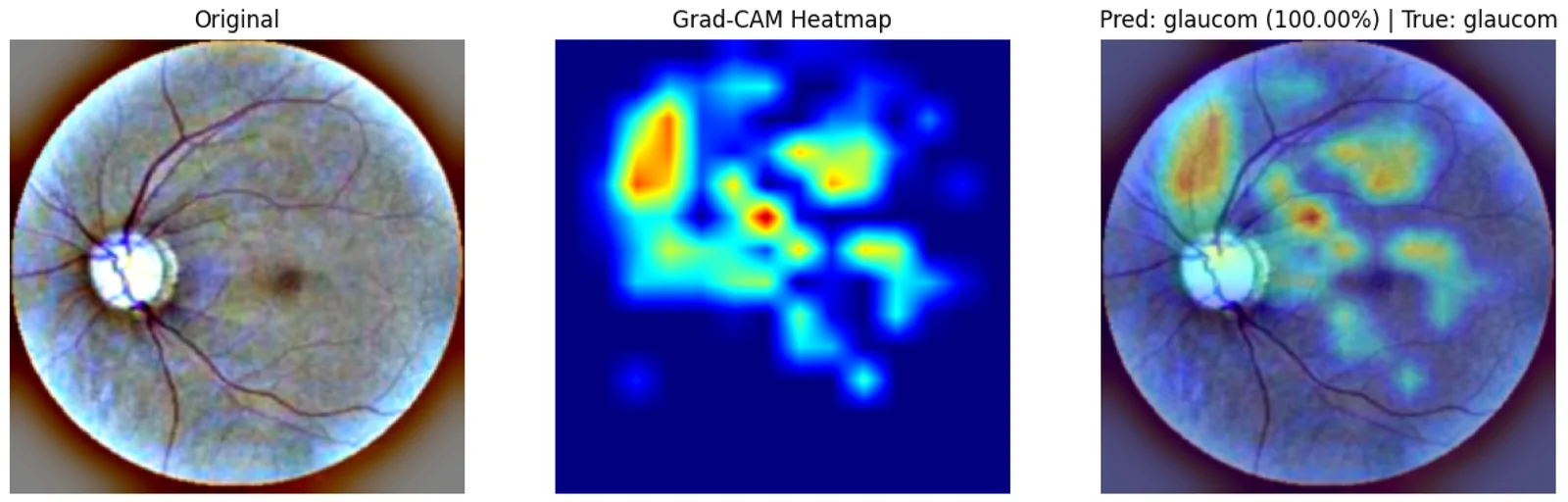
Grad-Cam related to an image belonging to the glaucoma class obtained with the model of Experiment 10.

**Figure 10:**
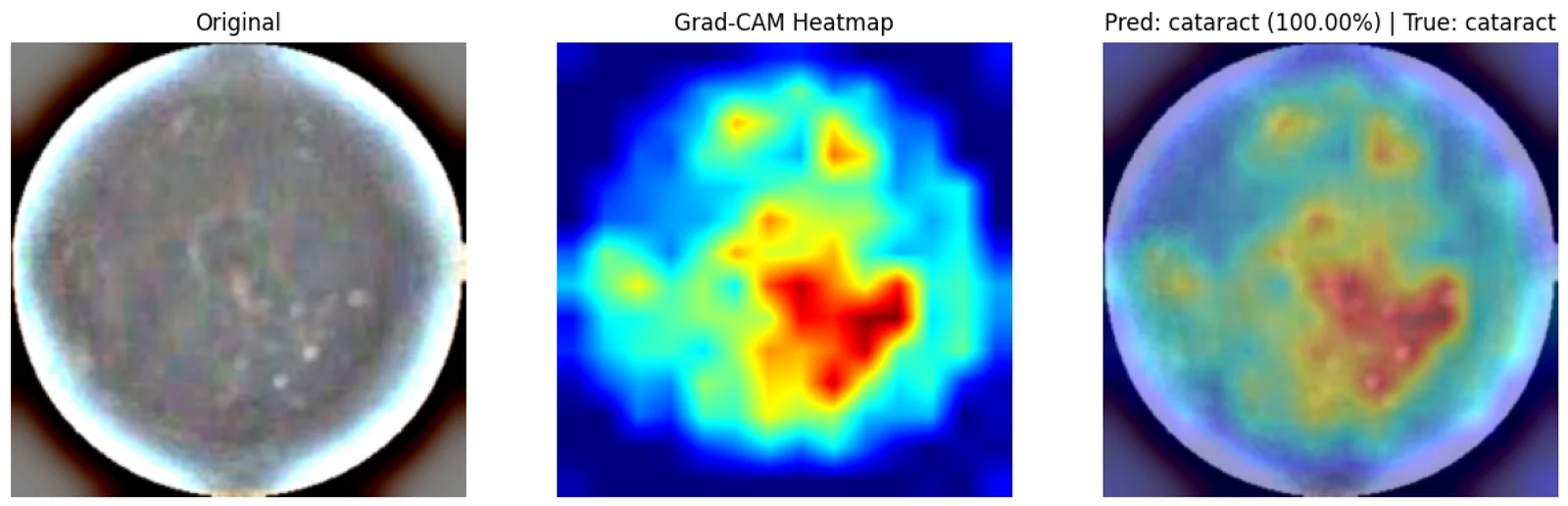
Grad-Cam related to an image belonging to the cataract class obtained with the model of Experiment 10.

In order to make the model’s decisions more transparent, the proposed method took into account the Grad-CAM technique. This method allowed us to overcome the limitation of the model being seen as a “black box”, as it generates a heatmap that highlights the areas that most influenced the classification, offering a visual interpretation of the model. Figure 5 presents the Grad-CAM obtained from the model of Experiment 3, applied to an image belonging to the normal class. It can be seen that the map is concentrated in the outer margin of the fundus, with particular attention to the area surrounding the optic disc and the main vessels. This activation indicates that the model learns to recognise the discriminative patterns of a healthy eye, where all the anatomical structures are present in their morphologically regular form, without alterations attributable to pathological conditions. Using the same model, obtained from Experiment 3, Figure 6 represents the Grad-CAM of an image belonging to the glaucoma class. The map is predominantly concentrated in the optic disc region and the peripapillary area; this behaviour is consistent with the pathology, as glaucoma is typically diagnosed by an increase in the cup-to-disc ratio (the ratio between the papillary excavation and the optic disc). Figure 7 shows the Grad-CAM of an image belonging to the cataract class obtained from the model in Experiment 3. Cataract is a disease of the lens that manifests itself as diffuse blurring, reducing visual quality. The Grad-CAM shown in Figure 7 reflects this phenomenon by showing a broad activation that tends to cover most of the visual field or to concentrate on the central areas of greatest opacity. After analysing the Grad-CAM heatmaps related to the experiment with the best metrics, attention was also turned to Experiment 10 which, despite performing slightly worse, showed good visual interpretability through the Grad-CAM technique. Figure 8, Figure 9 and Figure 10 show the activation maps for the normal, glaucoma and cataract classes, respectively, obtained from the model in Experiment 10. In Figure 8, the activation map shows a diffuse distribution, focusing on different retinal regions, including the vessels and the optic disc area. This behaviour is typical in the absence of localised pathological lesions; the model’s decision is based on overall anatomical characteristics rather than specific abnormalities. In Figure 9, which belongs to the glaucoma class, the heatmap is predominantly concentrated directly on the optic disc region and the immediately surrounding area. Figure 10, belonging to the cataract class, shows a general lack of sharpness and has a diffuse milky appearance typical of lens opacity. The Grad-CAM map is activated in the lower part of the lens where cortical opacity is predominant. Analysis of Grad-CAM maps demonstrates consistency between the regions highlighted by the model and the clinically relevant features of the various diseases considered. This technique provides a visual representation of the areas identified by the model as relevant for the discrimination and classification of fundus images. In this regard, the image regions activated during analysis can be used to support clinical diagnosis and enable specialists to conduct more in-depth investigations and monitor more frequently those patients who appear healthy but who present pathological patterns detectable by the model but not directly visible to the human eye.

## 4. Related Work

In this section we discuss the state of the art in the detection of eye diseases using DL. In the field of non-invasive ophthalmic diagnostic systems, a major challenge is the integration of biosensors into wearable devices. This is the context for the work of [7], who developed a hydrogel contact lens integrated with a multiplex diagnostic platform consisting of a passive RLC circuit inductively coupled to an external antenna. The system utilised electrical responses to monitor tear glucose levels, via changes in resistance across a graphene channel functionalised with the enzyme glucose oxidase and detected mechanical deformation of the cornea caused by intraocular pressure through changes in inductance and capacitance. Further advances related to these architectures have also been documented in the review by [8], where the aforementioned platforms were integrated with controlled-release and on-demand delivery systems designed to actively counteract the progression of glaucoma. Another important study conducted by [1] presented a federated analysis of a dataset consisting of 456 AngioOCT images and covering seven retinal diseases. The analysis involved the use of seven architectural configurations, including ViT, CNN, and hybrid models. Performance was evaluated in three classification scenarios: seven classes, four modified classes, and four simplified classes. Under controlled conditions, FL on simplified classes achieved performance levels of 0.72 in accuracy with FedAvg, FedProx, and Federated Magnetic Resonance Imaging (FedMRI), compared to 0.69 for centralised training. Full optimisation identified DenseNet121 as the optimal architecture, achieving an accuracy of 0.79 and ROC-AUC of 0.89. The task of detecting glaucoma using FL was also performed by [3]. This study involved seven clients located in hospitals in the USA, Hong Kong, and Singapore. Each centre first trained a model locally with its own set of OCT optic disc volumetric data, then uploaded the model parameters to the central server, which used the FedProx algorithm to aggregate the parameters. The FL model performed well with different networks in seven centres (accuracy of 0.78.3–0.98, 0.75–0.97, and 0.78–0.97.5) and stably in the two unseen datasets (accuracy of 0.85–0.87, 0.81.3–0.84.8, and 0.86–0.88, respectively). Collaborative model training between multiple entities without requiring data sharing or ensuring privacy protection was also adopted by [2], who conducted an analysis of DR severity based on FL. In order to identify signs of DR, five clients were considered who had different preprocessed fundus images from public datasets. The authors combined the FedAvg technique with the median of the categorical cross-entropy loss function and obtained an accuracy of 0.98, a specificity index of 0.99, a precision of 0.97, and an F1 score of 0.97. A further contribution was also made by [4], who conducted a binary classification study for the detection of glaucoma. The model was trained on a dataset divided into 60:20:20 proportions for training, validation and testing. The federated strategies adopted included FedAvg, FedProx, FedBN, and FedYogi; the experiment performed using FedProx achieved an accuracy of 0.76, a precision of 0.69, and a recall of 0.93. Fundus images analysis using DL was also proposed by [9], who trained a set of 2000 images with three DL models: Deep Neural Network (DNN), Feed Forward Neural Network (FNN) and CNN. In that case, the best accuracy was achieved by the DNN, at 0.89. The researchers in [10] considered 88,000 retinal photographs, obtaining an accuracy of 0.82 in detecting retinopathy and 0.51 in assessing its stage. The authors in [11], on the other hand, proposed a classification technique based on CNN but using images of the right and left eyes separately. The dataset used was preprocessed to improve the contrast between the images and to increase the volume of data. The best results were achieved using the VGG16 network, which achieved a specificity of 0.93, a sensitivity of 0.54 and an accuracy of 0.83. Another study was conducted by [12], who used two different public datasets, called MESSIDOR 2 and E-Ophtha, for a total of 75,137 images of the fundus. They considered a binary classification model (normal eye and eye affected by Diabetic Retinopathy); in this study, the accuracy was 0.97, the sensitivity index was 0.94, and the specificity reached 0.98. Gulshan et al. [13] also proposed an image classification method based on CNN training. Two Eye Picture Archiving and Communication System (eyePACS) datasets were used, for a total of 11,711 fundus images belonging to three distinct classes: Moderate Diabetic Retinopathy, Severe Diabetic Retinopathy, and Diabetic Macular Edema (DME). Pratt and his colleagues [14], on the other hand, proposed a CNN architecture aimed at classifying the different stages of DR; however, it is important to note that due to the nature of the architecture, the researchers were unable to accurately classify the mild stage of the disease. Specifically, on the dataset of 80,000 images used, the proposed CNN achieved a sensitivity of 0.95 and an accuracy of 0.75 on 5000 validation images. Neural networks have also been adopted for the classification of DR into three classes from authors in [15] that exploited characteristics such as the area of exudates, the area of blood vessels and texture parameters to classify images as normal, non-proliferative retinopathy and proliferative retinopathy. The neural network used these characteristics as input for classification, and the results obtained were validated and compared with the classification made by expert ophthalmologists. In particular, the study demonstrated an accuracy of 0.93 and a sensitivity of 0.90. As shown from the current state-of-the-art analysis, the presented studies do not consider the integration of explainability and privacy preserving approaches. In this scenario, the integration of FL with heatmaps introduces an effective method for the explainability of results and for the identification of discriminating patterns within the images under examination. Therefore, the proposed method provides an interesting contribution to the analysis of eye diseases through FL techniques and result explainability techniques using Grad-CAM explainability.

## 5. Conclusions and Future Work

In this paper we proposed a method for the automatic classification of fundus images for eye disease detection and localisation, which combined the adoption of FL, ViT and Grad-CAM, in order to address two very important challenges in the application of AI in the medical field (i.e., the security of sensitive patient data and the transparency of the model’s decision-making process). As a matter of fact, the use of FL enabled collaborative training between different clients without sharing sensitive data, as only model updates were transmitted to the central server, ensuring privacy while benefiting from the heterogeneity of multi-centre data. This is a very important factor in healthcare contexts, where restrictions on the sharing of patient data limit the development of robust and generalisable models. The use of the ViT-based classifier demonstrated solid performance in distinguishing between the three classes under consideration: normal eye, cataract, and glaucoma. The experimental analysis showed that both aggregation strategies, FedAvg and FedProx, achieved comparable results. The best configuration obtained in Experiment 3, based on the use of FedProx, achieved an accuracy of 0.8480, a precision of 0.8645, a recall of 0.8477 and an AUC of 0.9641. Although Experiment 10 conducted with FedAvg produced a significantly lower training loss, the FedProx-based model showed slightly better values in terms of precision and AUC. In support of the quantitative evaluation, the integration of the Grad-CAM explainability technique provided important qualitative evidence. The activation maps showed how the model consistently focused on clinically relevant regions, such as the optic disc in the case of glaucoma and diffuse opacity patterns in the case of cataract images. The consistency between where the model focuses its attention and medical diagnoses increases confidence in the reliability and explainability of the proposed method, by mitigating the so-called “black-box” limitation typical of DL models. The interesting results we obtained confirm that the combination of FL, ViT and explainability techniques represents a promising solution for eye diseases detection using ophthalmic image analysis.

For future work, several research directions can be explored to expand the proposed method. First, different federated aggregation strategies can be used, and further development could focus on expanding explainability. In addition to the Grad-CAM technique used in this work, other techniques such as attention rollout can be integrated. Finally, the adoption of quantitative explainability metrics could allow for a more rigorous assessment of the clinical consistency of the explanations generated. Specifically, the following indices could be considered: SSIM (Structural Similarity Index Measure), which assesses the perceptual similarity between two heatmaps in terms of luminance, contrast and structural alignment; VIF (Visual Information Fidelity), which measures the amount of visual information preserved between two heatmaps, using a wavelet-based statistical model of natural scenes; and finally, the SCC (Spatial Correlation Coefficient), which measures the local spatial correlation between activation maps. These indices can be estimated both to assess how different explainability techniques agree on the areas of the images identified as being of interest to a client’s model, and to estimate the agreement between activation maps generated by different clients.

## Figures and Tables

**Table 1:** Dataset distribution.

Set	Normal	Cataract	Glaucoma	Total
Training	350	350	349	1049
Validation	42	36	41	119
Testing	41	42	41	124

**Table 2:** Hyperparameter configuration adopted in the experimental phase for the 10 experiments.

Id	Clients	Epochs	Rounds	LR	Aggregation
1	3	5	80	0.0005	FedProx
2	5	12	45	0.0008	FedAvg
3	10	8	120	0.0007	FedProx
4	3	5	80	0.0005	FedAvg
5	5	12	45	0.0008	FedProx
6	10	8	120	0.0007	FedAvg
7	7	12	60	0.0005	FedProx
8	7	12	60	0.0005	FedAvg
9	10	8	100	0.0007	FedProx
10	10	8	100	0.0007	FedAvg

**Table 3:** Results obtained from the experiments.

Id	Accuracy	Precision	Recall	AUC	Loss
1	0.8240	0.8249	0.8244	0.9648	0.4422
2	0.8320	0.8349	0.8328	0.9548	0.3991
3	0.8480	0.8645	0.8477	0.9641	0.4134
4	0.8400	0.8393	0.8403	0.9648	0.4022
5	0.8000	0.8003	0.8006	0.9584	0.4078
6	0.8320	0.8646	0.8312	0.9600	0.4442
7	0.8240	0.8290	0.8248	0.9624	0.3866
8	0.8080	0.8060	0.8084	0.9542	0.3978
9	0.8160	0.8491	0.8153	0.9505	0.4720
10	0.8480	0.8599	0.8477	0.9590	0.3829

**Table 4:** Classes’ distribution for each client.

Client	Catarcat	Glaucoma	Normal
0	10	1	1
1	95	8	8
2	0	0	184
3	0	0	106
4	49	306	11
5	0	5	0
6	175	31	0
7	175	31	2
8	5	0	38
9	16	1	0

**Table 5:** Comparison among experiments.

Exp	Accuracy	Precision	Recall	AUC	Loss
IID	0.8480	0.8645	0.8477	0.9641	0.4134
non-IID	0.8000	0.8222	0.8008	0.9249	0.5923
Centralised	0.8080	0.8068	0.8080	0.9570	0.4079

## Data Availability

The original contributions presented in this study are included in the article. Further inquiries can be directed to the corresponding author(s).
